# Evolutionarily Conserved Histone Methylation Dynamics during Seed Life-Cycle Transitions

**DOI:** 10.1371/journal.pone.0051532

**Published:** 2012-12-11

**Authors:** Kerstin Müller, Daniel Bouyer, Arp Schnittger, Allison R. Kermode

**Affiliations:** 1 Biological Sciences, Simon Fraser University, Burnaby, British Colombia, Canada; 2 IBMP/CNRS - UPR2357, Strasbourg, France; Temasek Life Sciences Laboratory, Singapore

## Abstract

Plants have a remarkable ability to react to seasonal changes by synchronizing life-cycle transitions with environmental conditions. We addressed the question of how transcriptional re-programming occurs in response to an environmental cue that triggers the major life cycle transition from seed dormancy to germination and seedling growth. We elucidated an important mechanistic aspect of this process by following the chromatin dynamics of key regulatory genes with a focus on the two antagonistic marks, H3K4me3 and H3K27me3. Histone methylation patterns of major dormancy regulators changed during the transition to germination and seedling growth. We observed a switch from H3K4me3 and high transcription levels to silencing by the repressive H3K27me3 mark when dormancy was broken through exposure to moist chilling, underscoring that a functional PRC2 complex is necessary for this transition. Moreover, this reciprocal regulation by H3K4me3 and H3K27me3 is evolutionarily conserved from gymnosperms to angiosperms.

## Introduction

Our current knowledge about histone methylation depicts a rather static picture. Moreover, with the exception of a few developmental regulators involved in flowering induction [1], [2], little is known about the dynamics of epigenetic regulation during phase transitions in plants. The plant life cycle is characterized by major transitions in response to integrated internal and environmental cues that require extensive reprogramming of the transcriptome. For seed plants, the transition from a dormant to a non-dormant state of the seed is of major importance for the plant’s success in establishing a new generation. Seed dormancy is defined as the inability of a viable seed to complete germination under favorable conditions [3], [4]; the transition to germination requires a specific environmental trigger. Transcriptomic analyses reveal strict spatial and temporal regulation of gene expression during the dormancy-to-germination transition [5], [6]. However, it is largely unknown how the major dormancy regulators are themselves regulated in response to environmental cues.

DNA in the eukaryotic nucleus is organized to form chromatin. The minimal unit, the nucleosome, is composed of the DNA wrapped around complexes of eight histone cores. Histones can be modified at their N-terminus at a number of residues and with different modifications [7]. Methylation of histones is one mechanism that contributes to the spatial and temporal regulation of gene expression on which growth and development depends in all eukaryotes [8], [9]. Depending on the amino acid residue that is modified and the number of methyl groups added to the residue, histone methylation changes (‘marks’) can contribute to transcriptional activation or repression. For example, trimethylation of histone H3 lysine 4 (H3K4me3) is an ‘activating mark’, while trimethylation of lysine 27 of histone 3 (H3K27me3) leads to repression of transcription [7], [8].

Histone modifications are important in regulating various life cycle transitions in plants, including the transition from vegetative to reproductive growth in flowering plants [10], [11], and from gametophyte to sporophyte development in the moss *Physcomitrella patens* [12]. To date chromatin modifications have not been explored in mature seeds.

Mutants in the polycomb repressive complex 2 (PRC2) display a significant delay in germination and express markers of seed development and dormancy in seedlings [13], leading to the hypothesis that histone methylation pathways play a role in the transition from dormancy to germination and seedling growth. PRC2 acts as a histone methyltransferase (HMTase) on lysine 27 of histone 3 (H3K27me3) leading to gene silencing. Moreover, the Arabidopsis *pickle* (*pkl*) mutant, which is affected in H3K27me3 deposition [14], [15], exhibits germination-associated phenotypes and expresses markers associated with seed development and dormancy during seedling growth [15].

In this work, we elucidated an important mechanistic aspect underlying the global transcriptional network re-programming during seed dormancy breakage and the transition to germination. This was achieved by following the chromatin dynamics of key regulatory genes with a focus on the two antagonistic marks, H3K4me3 and H3K27me3.

**Figure 1:**
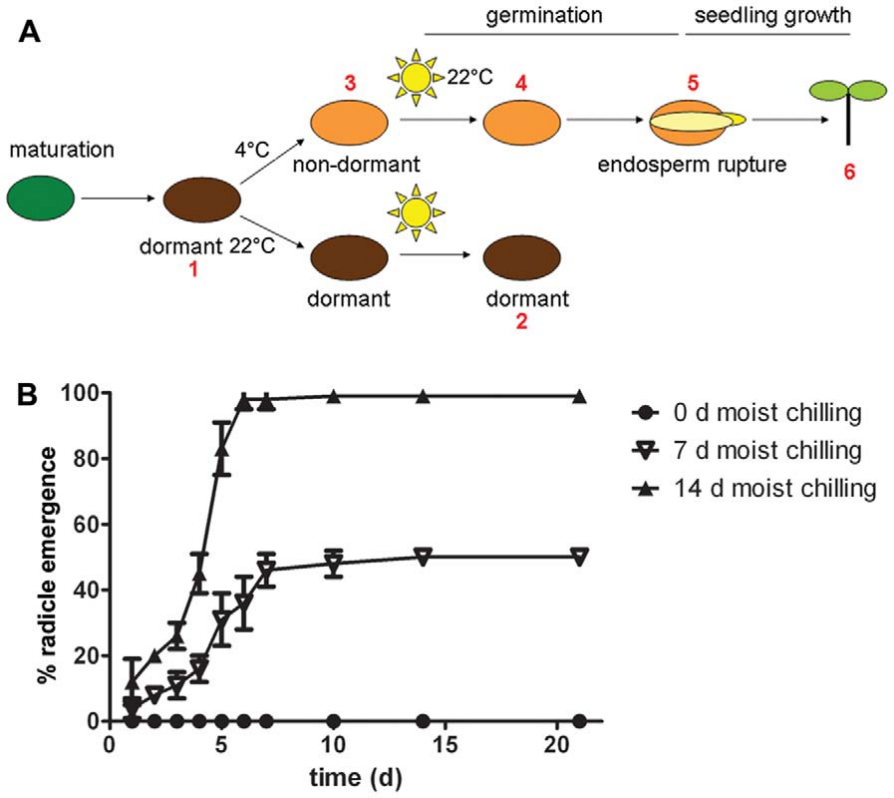
Schematic representation of Arabidopsis Cvi seed treatments and physiological status of the seeds. (**A**) The inception of primary seed dormancy occurs during seed maturation. Mature dry seeds are dormant (*1*) and are maintained in this state when imbibed at 22°C, even for 14 d (*2*). A sufficiently long period of moist chilling (4°C) will break dormancy (*3*). Light (sun symbol) can induce germination at 22°C once dormancy is broken by moist chilling – seeds commence germination (*4*), which proceeds to radicle protrusion, signifying the completion of germination (*5*). The next transition is from germination to seedling growth/development (*6*). (**B**) Characterization of seed dormancy of Arabidopsis ecotype Cvi. Moist chilling is required for 14 d to subsequently elicit the full germination potential of seeds. Data are based on mean values of three replicates of 50 seeds +/− SE.

## Materials and Methods

### Seed Materials, Dormancy-Breaking and Control Treatments, and Germination Testing

*Arabidopsis thaliana* ecotype Cvi plants were grown in a growth chamber at 22°C in soil under 16-h photoperiod. Mature dry seeds were harvested and surface-sterilized with 70% and 100% ethanol. Seeds were either monitored for their germination capacity immediately (no moist chilling), or were first moist-chilled at 4°C in the dark on half-strength Murashige and Skoog (MS) media (pH 6.5) solidified with 1% agar for 7 and 14 d prior to germination testing. For germination tests, 3×50 seeds were sown on plates containing solid half-strength MS media (pH 6.5) and the plates were transferred to a Conviron CMP3244 growth chamber (22°C, 16-h photoperiod). Seeds were scored as germinated based on radicle emergence with the aid of a dissection microscope. A control treatment for the moist chilling was conducted in which seeds were sown on plates containing solid half-strength MS media and kept at 22°C under long day conditions for 14 d.

**Figure 2:**
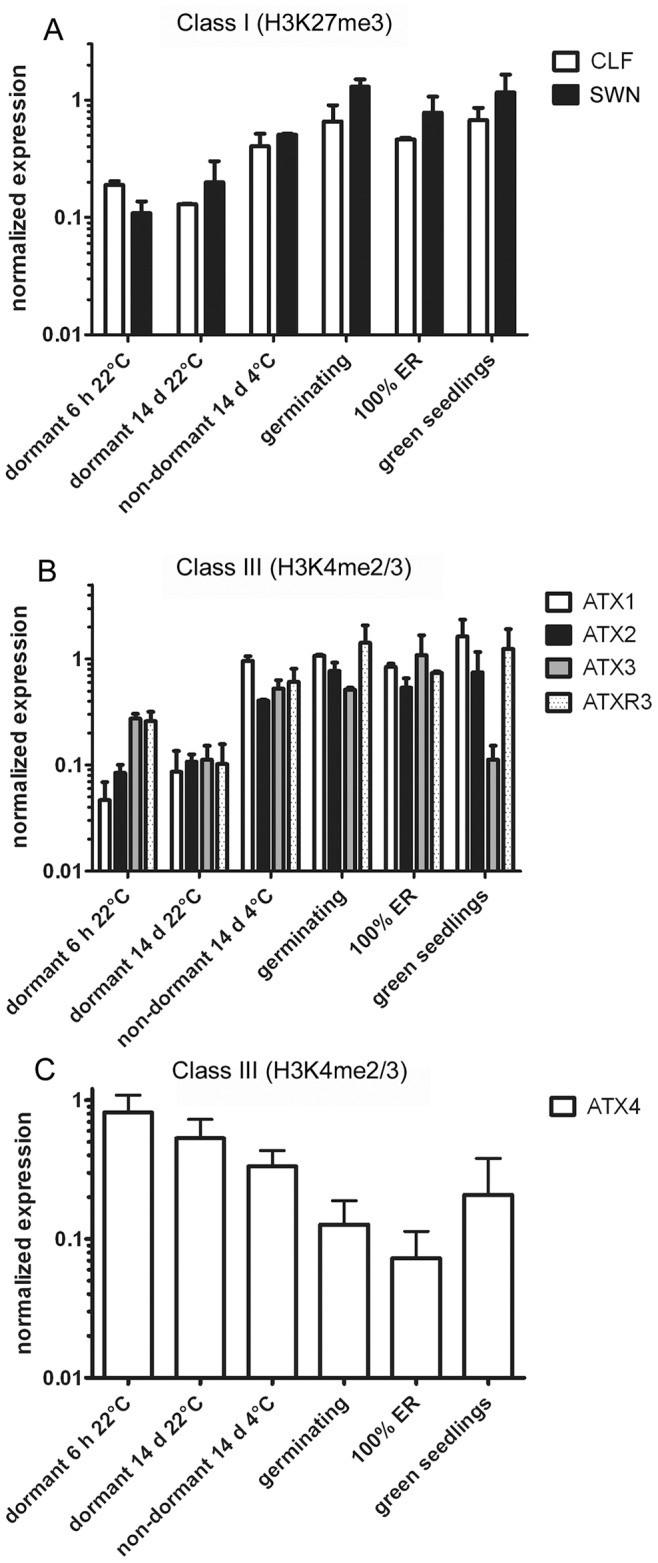
Expression analyses of histone methyltransferases (HMTs) in Arabidopsis Cvi seeds. (**A**) Class I SET-domain HMTs. (**B, C**) Class III SET-domain HMTs. Mean of three biological replicates +/− SE are shown. Note that the Y-axis for the RNA data is in log 10-scale.

Yellow-cedar seeds (seedlot 48827) were obtained from the BC Ministry of Forests and Range Tree Seed Centre (Surrey, BC, Canada). The conditions for dormancy termination and germination were as described [16]. In brief, the full dormancy-breaking treatment included treating seeds first with a 3-d running water soak (22±1°C), followed by a four-week warm, moist period in which seeds were placed in seed boxes on filter paper soaked with water and maintained in darkness at 25°C. Seeds were then moist-chilled for 8 weeks (at 4°C in darkness). For germination, seeds were transferred to a controlled incubator (16 h light, 30°C; 8 h dark, 20°C). A control treatment consisted of the 3-day soak followed by 12 weeks of warm, moist conditions (darkness, 25°C). This control treatment did not break dormancy.

**Figure 3:**
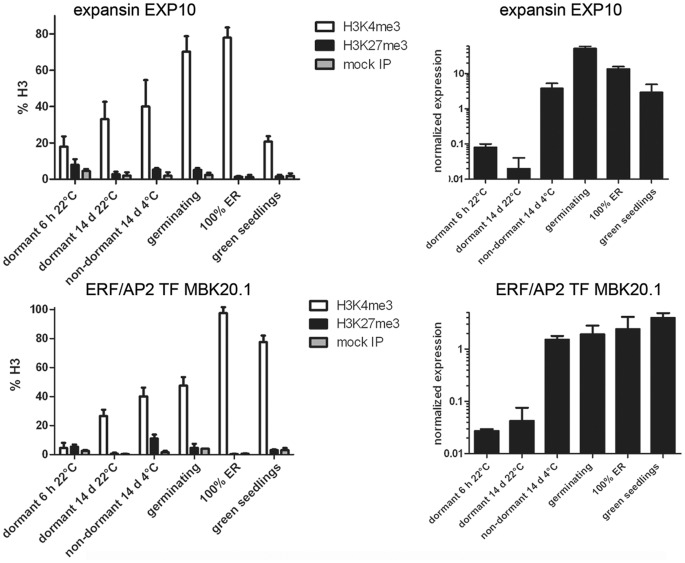
Expression analyses and histone H3 methylation pattern changes of germination-associated genes in Arabidopsis Cvi. nChIP/qPCR (left column) and expression analyses (right column); averages of three biological replicates are shown +/− SE. ER = endosperm rupture (completion of germination). Note that the Y-axis for the RNA data is in log-scale.

### Target Genes

The target genes for this study are noted in Table 1. The TAIR numbers for these genes were as follows: *ABI3* (At3g24650), *LEC2* (At1g28300), *DOG1* (At5g45830), *SOM* (At1g03790), *RAB18* (At5g66400), *2S1* (At4g27140), *FLC* (At5g10140), *MBK20.1* (At5g07580), *COR 47* (At1g20440), *EXP10* (At1g26770), *MDAR6* (At1g63940), *PBC2* (At1g77440), *HB1* (At3g01470).

**Figure 4:**
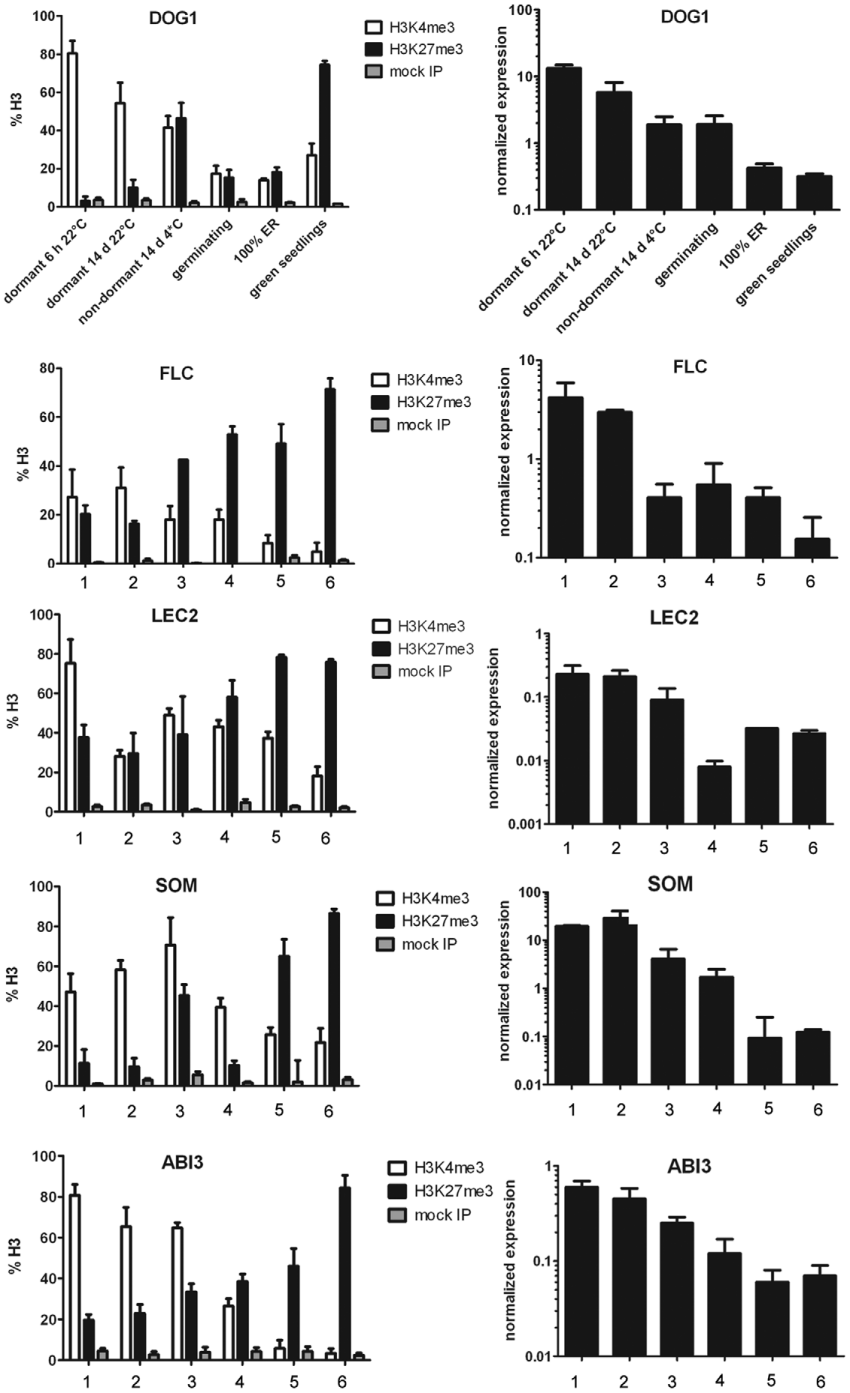
Expression analyses and histone H3 methylation pattern changes of regulators of seed maturation/dormancy in Arabidopsis Cvi. nChIP/qPCR (left column) and expression analyses (right column); averages of three biological replicates are shown +/− SE. Numbers 1–6 correspond to the stages noted on the X-axis for DOG1. ER = endosperm rupture (completion of germination). Note that the Y-axis for the RNA data is in log-scale.

### Seed Samples

For ChIP and RNA extractions, three replicates of 100 mg of Arabidopsis seeds were used for each treatment. After the described treatment, each seed batch was aliquoted equally into two tubes, flash frozen in liquid nitrogen and stored at −80°C until use. One tube was used for RNA extraction and the other for ChIP. For yellow-cedar samples, megagametophytes and embryos were excised with a scalpel, flash frozen in liquid nitrogen and stored at −80°C until use.

**Figure 5:**
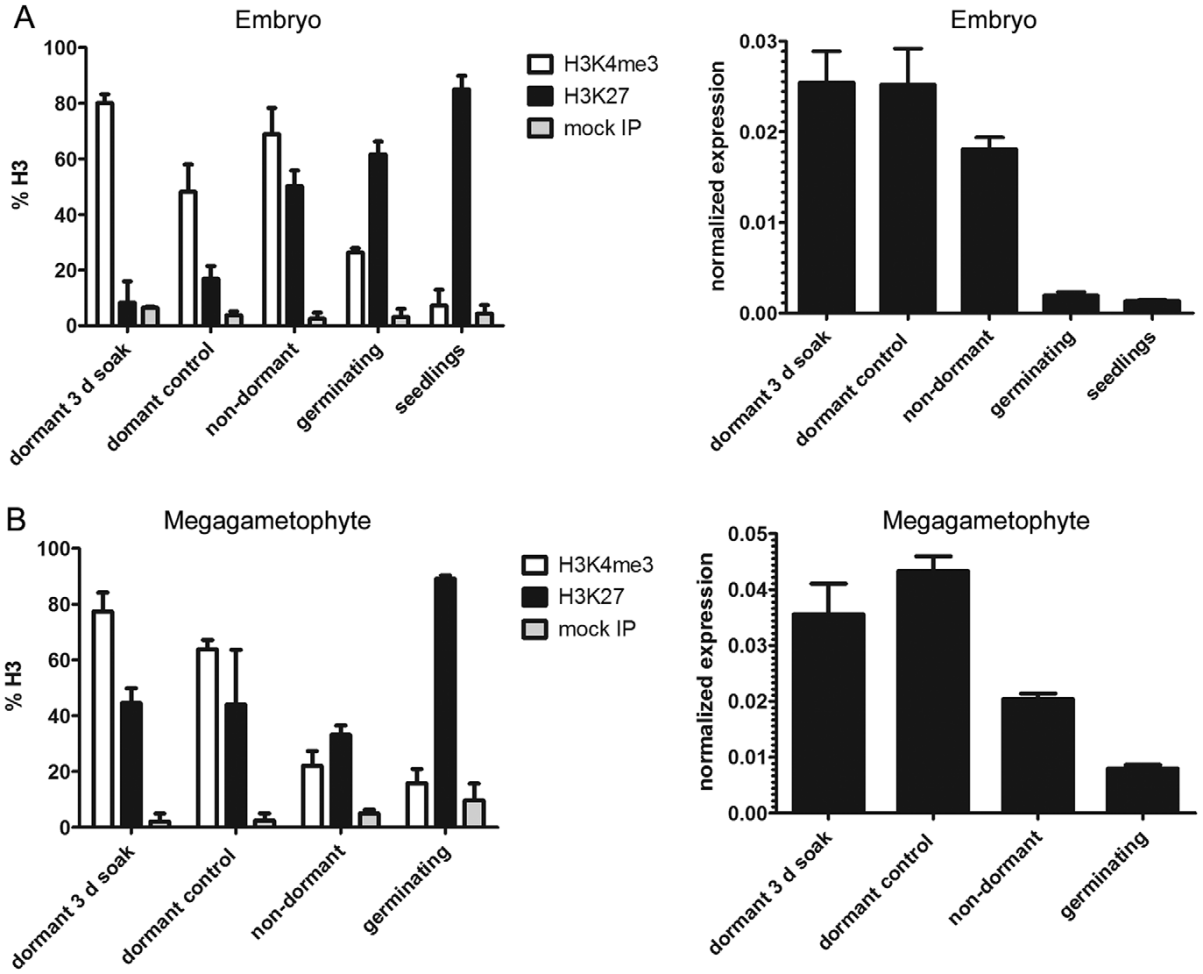
Expression analyses and histone H3 methylation pattern changes of *CnABI3* in yellow-cedar. (**A**) Embryos (**B**) Megagametophytes. nChIP/qPCR (left column) and expression analyses (right column). Averages of three biological replicates are shown +/− SE. Mature yellow-cedar seeds were subjected to a full (12-week) dormancy-breaking treatment, or the full dormancy-breaking treatment followed by 1 day in germination conditions (“germinating”). Seedlings were at an early stage (4 mm radicle length, and greened cotyledons). A control treatment consisted of maintaining seeds in warm, moist conditions for 12 weeks, which did not break dormancy.

**Figure 6:**
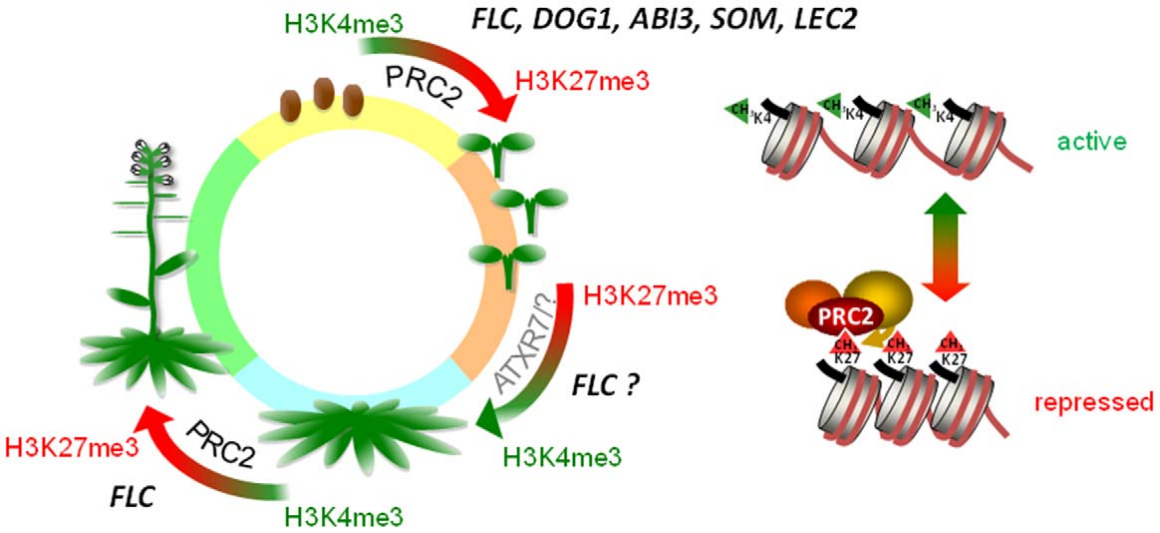
Model illustrating the essential role of histone modifications during seed-to-seedling and vegetative-to-generative phase transitions. Repressive H3K27me3 marks on genes encoding key regulators of dormancy, such as *ABI3*, are deposited through the PRC2 complex, and replace the activating H3K4me3 mark in response to the environmental cue of moist chilling, similar to the vernalization response on the FLC locus. In order to fulfill its role as a flowering repressor, the FLC locus has to be re-activated after seed germination. ATXR7 might be involved in this process [46].

### RNA Extraction and cDNA Synthesis

Whole seeds of Arabidopsis, yellow-cedar embryos and yellow-cedar megagametophytes were ground in liquid nitrogen and total RNA was extracted as described [17] with the following modifications. After addition of CTAB buffer (2% hexadecyl trimethyl-ammonium bromide (CTAB), 2% polyvinylpyrrolidone [MW = 40000/K30], 100 mM Tris-HCl, pH 8.0, 25 mM EDTA, pH 8.0, 2 M NaCl, 2% ß-mercaptoethanol), the extracts were kept at 65°C for 10 min. All chloroform:isoamyl alcohol extractions were repeated once. RNA was treated with DNase-I (Fermentas) to remove remaining genomic DNA and the integrity of the RNA was checked on an agarose gel followed by quantity and purity determination with a nanodrop spectrophotometer (ND-2000C, Thermo Scientific). One µg RNA was reverse transcribed using the EasyScript Plus kit (abmgood) with a mixture of random hexamers and oligo-dT primers. cDNA from three biological replicate RNA samples was used for qPCR.

### Quantitative (q)RT-PCR

qRT-PCRs were run in 15 µl reaction volumes on an ABI7900HT machine (Applied Biosystems) using the PerfeCTa Sybr Green Supermix with ROX (Qanta Biosciences). Primers (Table S1) were designed with the primer3 [18] tool in Geneious 4.8.5 and based on published sequences. At1g17210 and At2g20000 [19] were used as reference genes. As the published sequences had been obtained using other ecotypes, all primers were tested on Cvi cDNA before being used in the PCRs. The reaction mixture consisted of 150 ng cDNA (RNA equivalent), 7.5 µl supermix and 140 nM of each primer, and the temperature regime was 3 min at 95°C and 40 cycles of 15 s at 95°C/1 min at 60°C. A dissociation curve was run after each qPCR to validate that only one product had been amplified.

The efficiency E of the primer pairs was calculated as the average of the Es of the individual reactions by using raw fluorescence data with the publicly available PCR Miner tool [20]. The efficiency was then used to calculate transcript abundance for the individual samples as (1+E)^∧^ (−CT). No-template-controls (NTCs) for each primer pair were included to check for contamination of the reagents. Only samples whose corresponding NTC showed no amplification signal were used in the analysis.

### Native ChIP (nChIP) for Seeds and Seedlings

Samples were ground in liquid nitrogen, and added to 15 ml nuclei enrichment buffer (150 mM sucrose, 10 mM Tris pH 7.2, 5 mM MgCl_2_, 5 mM beta-mercaptoethanol, 20% glycerol, 1×protease inhibitor complex) (PIC, abmgood) on ice. The suspension was filtered through a double layer of miracloth and Triton X-100 was added to a final concentration of 1%. After rotation at 4°C for 10 min and centrifugation at 4000 g for 10 min at4°C, the pellet was resuspended in 300 µl nuclease buffer (30 mM Tris pH 8.5, 2 mM Mg-acetate, 4% glycerol, PIC) for micrococcal nuclease digestion. For nChIP of isolated Arabidopsis embryos, 300 embryos were carefully dissected from enclosing seed tissues, ground in liquid nitrogen and transferred directly to nuclease buffer. Micrococcal nuclease (2.5 µl) (Worthington Biochemicals) was added and the sample incubated for 8 min at 37°C. The reaction was stopped with EDTA (final concentration 10 mM) and the nuclei lysed by adding 900 µl lysis buffer (30 mM Tris pH 8.0, 2 mM DTT, 150 mM KCl, 0.5% Triton X-100, PIC), vortexing for 1 min, incubating the tubes on ice for 10 min, followed by vortexing for 1 min. Samples were centrifuged for 2 min at 4000 rpm at 4°C and the supernatant transferred to new tubes. The sample were pre-cleared with 30 µl salmon-sperm DNA- and BSA-blocked A/G beads at 4°C for 2 h on a rotating wheel. After centrifugation at 4°C, 4000 rpm, the supernatant was collected. This is the pre-cleared chromatin sample.

Pre-cleared chromatin (250 µl) was used per immunoprecipitation (IP). One hundred µl of the sample was used as input and left at 4°C overnight. Chromatin was immunoprecipitated with antibodies against unmodified histone H3 (Sigma Aldrich, H9289), H3K4me3 (Abcam, 8580) and H3K27me3 (Abcam, 6002), respectively. As a negative control, a mock IP with IgG (Sigma Aldrich) was performed. Thirty µl blocked A/G beads were added to each reaction and the samples rotated at 4°C overnight. The beads were then spun down and washed twice with 400 µl wash buffer (20 mM Tris/HCl pH 8.0, 0.1% SDS, 1% Triton X-100, 2 mM EDTA, PIC), containing 150 mM NaCl for 5 min at 4°C, and once with 400 µl wash buffer containing 500 mM NaCl. After the last centrifugation, the supernatant was discarded and 100 µl elution buffer (100 mM NaHCO_3_, 1% SDS) was added to all samples including the untreated input sample. All samples were incubated at 65°C for 10 min, centrifuged at 4000 rpm for 1 min at room temperature and the supernatants were collected. The elution step was repeated and the eluates pooled.

### Purification of ChIP Eluates

Ten µg RNAase A (Fermentas) was added to the eluates, and the mixture was incubated for 30 min at room temperature. DNA was purified with a nucleic acid purification kit (Epoch Lifescience) and eluted with 20 µl elution buffer. The resulting DNA solution was diluted five times and 2 µl was used as a template for qPCR.

### Native ChIP of Yellow-Cedar Seed and Seedling Tissues

For yellow-cedar embryos and megagametophytes as well as seedling nChIP, 60 seed parts or 20 seedlings underwent the same protocol as described for Arabidopsis in Material and Methods, with one modification. In the washing step after the IP, beads were washed twice for 10 min in low-salt wash buffer and twice for 5 min in high-salt wash buffer.

### Native ChIP/qPCR

qPCRs using purified nChIP-DNA as a template were run in 15 µl reactions on an ABI7900HT machine with EvaGreen qPCR mastermix with ROX (abmgood). Primers (Table S1) were placed in the regions in which the UCSC Genome Browser (http://epigenomics.mcdb.ucla.edu, 21) indicated the presence of the marks in seedlings. The reaction mixture consisted of 2 µl diluted ChIP-DNA (4 µl for yellow-cedar), 7.5 µl mastermix, and 280 nM of each primer, and the temperature regime was 10 min at 95°C and 40 cycles of 15 s at 95°C/1 min at 60°C followed by a dissociation curve. Template abundance for individual samples was calculated as described above. For the comparison of different samples, the IPs of modified histones were normalized to the IPs of unmodified H3 to account for possible variability in histone density.

## Results and Discussion

### Activation of Germination Regulators by H3K4me3 during the Dormancy-to-germination Transition in ARABIDOPSIS Cvi

The Arabidopsis ecotype *Cape Verde Island* (Cvi) is a model for seed dormancy, and its transcriptomic changes during dormancy maintenance and termination have been characterized [5], [6]. The treatments used in the present study to capture key stages of the dormancy-to-germination transition and the corresponding general physiological status of the Cvi seeds are illustrated in Fig. 1A. Freshly harvested mature seeds were dormant, i.e. they did not germinate when placed in germination conditions, unless first subjected to moist chilling (Fig. 1B). 14 d of moist chilling was required to break dormancy and elicit 100% germination. The control-treatment (warm, moist conditions for 14 d) did not break dormancy and none of the seeds germinated. The actual environmental cue that breaks the dormancy of the mature imbibed seed is thus extensive cold temperature.

Given the evidence for chromatin changes during other plant lifecycle transitions (see Introduction), and the observation that *prc2* mutants of Arabidopsis display enhanced dormancy [13], we hypothesized that a tight regulation of chromatin changes is crucial for gene regulation during seed development, dormancy and germination. Therefore, we established a protocol for native chromatin immunoprecipitation (nChIP) for seed material. nChIP does not involve chemical crosslinking of protein and DNA, but instead relies on the strong attachment of the DNA to the histones in the nucleosome and isolation of nucleosomal fragments by micrococcal nuclease digestion. nChIP has been recently adopted for Arabidopsis vegetative tissues, and reveals a highly similar pattern compared to formaldehyde-fixed material in H3K9me2 profiling [22].

For our analyses we focused on two key histone marks: H3K4me3, which is associated with transcriptionally active genes, and H3K27me3, an inhibitory mark. The deposition of both marks is mediated by trxG- and PcG SET domain proteins respectively [23], and the corresponding HMTases showed differential expression patterns during the dormancy-to-germination transition, suggesting that changes in the chromatin landscape take place (Fig. 2).

We investigated a range of genes that encode proteins with a demonstrated or intimated positive regulatory role in seed maturation and/or dormancy, as well as genes encoding proteins that can be classified as markers of seed dormancy (Table 1). For markers and regulators of germination, we selected six genes that exhibit the strongest up-regulation during dormancy termination of Cvi seeds based on published transcriptomes (Table 1) [5], [6]. These germination-associated genes bear the H3K4me3 in seedlings, and we asked whether this histone methylation is deposited during the actual dormancy-to-germination transition (Figs. 3, S1). Indeed, all six genes showed increasing amounts of H3K4me3 from the dormant seed to the seedling stage. In contrast, these genes were generally not decorated with H3K27me3. One exception was the dehydrin *COR47*, which carried H3K27me3 in the dormant state. This repressive mark on *COR47* was gradually lost and exchanged for increasing amounts of H3K4me3 following transfer of seeds to germination conditions (Fig. S1) Therefore the transcriptional activation during germination is consistent with an extensive reprogramming at the chromatin level.

### Histone Methylation Dynamics of Major Seed Maturation- and Dormancy-regulators during the Environmentally Cued Transition from Dormancy to Germination

Our new nChIP protocol allowed us to focus on the dynamics of major dormancy regulators in seeds at key physiological stages (Fig. 1). We decided to follow seven central regulators and markers (Table 1): The gene products of *ABI3* (24) and *LEC2* [25], [26] control seed maturation and dormancy [4], [27]. *Delay of Germination1* (*DOG1*) is a major dormancy QTL in Arabidopsis [27], [28]. SOMNUS (*SOM*) positively influences signaling of the dormancy-inducing and germination-inhibiting plant hormone abscisic acid (ABA) and negatively influences signaling of its antagonist gibberellin (GA) [29]. *FLC* has been implicated in the regulation of temperature dependent seed germination [30], but is best known for its role as a repressor of flowering, in which context it is subject to epigenetic regulation when plants are exposed to cold temperatures during vernalization [1], [2]. Our analyses also included the gene for the storage protein 2S1 (a maturation marker) as well as *RAB18*. RAB18 likely plays an indirect role in dormancy, as this protein is connected more to the survival of the seed in the dispersed, dormant state (e.g. tolerance of the dispersed seed to environmental stresses such as water/desiccation stress) (Table 1). The *RAB18* gene is expressed during late seed maturation, and exhibits reduced expression during germination. Therefore it is most accurately considered a “dormancy marker”, and not a dormancy regulator.

Major changes in transcript abundance of the genes encoding regulators and markers of seed maturation and/or dormancy occurred during dormancy-termination *per se* (e.g. *DOG1* and *FLC*), or once germination had been induced (e.g. *ABI3* and *LEC2*) (Fig. 4). *SOM* expression was most strongly down-regulated upon the completion of germination (Fig. 4). The “marker” genes, *RAB18* and *2S1*, showed the greatest decline in abundance during germination (Fig. S2).

The switch from activating H3K4me3- to repressive H3K27me3-deposition was associated with a change in transcript level of the dormancy regulators (Fig. 4). We are thus able to discriminate between genes that are required for germination and genes involved in dormancy by their H3 methylation patterns. The former show a strong transcriptional up-regulation during germination that is associated with H3K4me3 deposition. This mark seems to be stable throughout further development and growth as it is also found in genome-wide H3K4me3 profiling studies using 10–20 day old seedlings [13], [31], [32]. The dormancy regulators were found to maintain H3K27me3 throughout the subsequent seedling stage [13], [33], [34]. The transition to another life phase is directly reflected in a change at the chromatin level that is then maintained throughout further development. The cue for this life-cycle transition is the exposure of the imbibed seeds to low temperatures. The environmental temperature signal is therefore transduced to effect the observed chromatin changes. It is of interest to investigate whether the same patterns of histone modifications are transduced by other cues that effectively break seed dormancy such as afterripening.

*FLC* deviates from the general pattern of a maintenance of repressive marks throughout the rest of the life cycle. Although this gene also showed a replacement of H3K4me3 by H3K27me3 during seed dormancy release by moist chilling and germination, *FLC* must be reset to an active state very soon after germination to fulfill its role as a negative regulator of flowering. However *FLC* has been tested both positive and negative for H3K27me3 in Arabidopsis plants, depending on natural variation, developmental state, and possibly growth conditions, respectively [28], [34], [35]. Recent work by R.R. de Casas et al. [36] shows that moist chilling of seeds leads to earlier flowering in the resulting plants independently of the dormancy status of the seeds. It is thus possible that the appearance of H3K27me3 on *FLC* is caused by exposure to low temperatures, and not by the physiological process of dormancy breakage *per se*. The exposure of seeds to moist chilling might thereby lead to *FLC* repression on the chromatin level such that earlier flowering is promoted in the adult plants.

A. Angel et al. [37] have described a nucleation process that takes place on the *FLC-*locus during induction of flowering competence through vernalization: H3K27me3 accumulates slowly over weeks of cold exposure in one segment of the *FLC* gene in the sampling population. When plants are returned to warm conditions, the mark spreads over the whole gene depending on the length of period of cold exposure, and the presence of the mark is quantitatively correlated with *FLC* expression [37]. Moreover, the quantity of initial H3K27me3 deposition and spreading over the gene body is linked to polymorphisms at the cis-level that reflects the different need for cold temperature exposure in different accessions [35]. The required period of vernalization (for Arabidopsis accessions requiring this cue to trigger flowering) is typically much longer that the moist chilling period required for Arabidopsis seed dormancy breakage. Yet the similarities between the two processes are striking. It is thus interesting to speculate that a similar polymorphism-based mechanism is the underlying basis for some of the variations in cold-requirements for dormancy breakage, and for the huge variation in the depth of seed dormancy. The mechanisms observed in the context of vernalization effects at the chromatin level certainly seem comparable to the gradual accumulation of H3K27me3 and simultaneous reduction of H3K4me3 that we observed on seed dormancy regulators during moist chilling of seeds. It is conceivable that germination *competence* is reached at a certain threshold ratio of active to repressive marks on dormancy regulators, and that germination proceeds only when genes specifying positive germination regulators are activated. This would provide a quantitative means to measure the extent of cold exposure as a result of the output of the ratio of activating and repressive marks.

Interestingly, we detected both histone marks at the corresponding loci for maturation/dormancy-related genes in our sampling population in non-dormant seeds before their transfer to germination conditions (Figs. 4 and S2). H3K27me3 thus gradually replaces H3K4me3 on the major dormancy regulators, until no or very little H3K4me3 remains detectable in seedlings. It is however unlikely that these would represent so-called bivalent marks, which are found in mammalian embryonic stem cells [38], as evidence for this is scarce in plants [39].

Very little is known about the mechanistic aspects of the interactions between different histone modifications in plants. By combining our findings with H3K4me3 profiling in *prc2* mutants [13], we found that this mark needs to be replaced by H3K27me3 in order to be removed as many of the dormancy regulators that we investigated such as *ABI3*, *DOG1*, and *FLC*, still show H3K4me3 in seedlings upon loss of H3K27me3 [13] (Fig. S3). This is remarkable as only a small portion of PRC2-target genes in the genome show this gain in H3K4me3 upon loss of PRC2, suggesting that its activity is necessary to replace the activating mark and effect the termination of gene expression.

PRC2-mediated dormancy control appears to take place at the level of the embryo, as seeds with homozygous *PRC2*-defective endosperm but heterozygous embryos exhibit germination behavior that is indistinguishable from that of wild-type seeds [13]. Therefore, we expanded our nChIP analyses to isolated embryos from seeds that had been exposed to the dormancy-terminating treatment (14 d of moist chilling). The histone profiles of the embryos strongly resembled those of whole seeds (Fig. S4). Therefore the dynamic change from the activating to the repressive state very likely takes place in the embryo during dormancy breakage.

### Changes in Histone Methylation of the *ABI3* Gene, a Major Regulator of Life Cycle Transitions, are Evolutionarily Conserved

Having found that major dormancy regulators such as ABI3 are transcriptionally regulated at the chromatin level in Arabidopsis, we asked whether the same is true for an evolutionarily distant species, the gymnosperm yellow-cedar (*Callitropsis nootkatensis*). The seeds of this conifer species are deeply dormant at maturity and upon dispersal they typically require a minimum of 6 months of moist chilling in natural stands to terminate dormancy [40]. There is a marked conservation of the functions of the ABI3 orthologs of evolutionarily distant species, including angiosperms, conifers and even mosses [27], [41]–[43]. Similar to its angiosperm counterparts, the yellow-cedar ABI3 (CnABI3) functions in maturation processes and is a positive regulator of dormancy [41], [44].

In both the yellow-cedar embryo and the megagametophyte storage tissue we found the same regulation of *ABI3* on the chromatin level in yellow-cedar seeds as that within Arabidopsis seeds: a shift from H3K4me3 to H3K27me3 occurred during the dormancy-to-germination transition, and this shift was associated with transcriptional repression (Fig. 5).

ABI3 proteins are known to play a role as a ‘gatekeeper’ of various life-cycle transitions [45]. The commonalities of the epigenetic transcriptional regulation of the *ABI3* gene indicate that this major regulator of life-cycle transitions is subject to evolutionarily conserved regulatory mechanisms. This conservation between gymnosperms and angiosperms suggests that the regulation of expression of central dormancy regulators by histone modifications was likely established very early in the evolution of seed plants.

### Conclusions

In conclusion, we propose that H3K27me3 deposition through the PRC2 complex is necessary to replace the activating mark H3K4me3 and repress the expression of dormancy-related genes (Fig. 6) upon dormancy termination (elicited by moist chilling) and germination. Our model further asserts that once a threshold level of repressive marks is reached, the seeds become competent to germinate; induction of the process of germination that occurs when the seeds are placed in favorable conditions is accompanied by the activation of transcription of ‘germination/growth’ genes via the accumulation of H3K4me3. Thus the reprogramming of the chromatin state plays an essential role in the integration of internal and environmental cues by seeds, thus permitting the transition to the next life phase.

## Supporting Information

Figure S1 **Expression analyses and histone H3 methylation pattern changes of regulators and markers of seed germination in Arabidopsis Cvi.** Supplementary results to support data of **Fig. 3** **.** nChIP/qPCR (left column) and expression analyses (right column); averages of three biological replicates are shown +/− SE. Refer to Table 1. ER = endosperm rupture and radicle emergence (completion of germination). Note that the Y-axis for the RNA data is in log-scale.
(JPG)

Figure S2 **Expression analyses and histone H3 methylation pattern changes of markers of seed maturation/dormancy (** ***2S1*** **and** ***RAB18*** **) in Arabidopsis Cvi.** Supplementary results to support data of **Fig. 4** **.** nChIP/qPCR (left column) and expression analyses (right column); averages of three biological replicates are shown +/− SE. Refer to Table 1. ER = endosperm rupture and radicle emergence (completion of germination). Note that the Y-axis for the RNA data is in log-scale.
(JPG)

Figure S3 **Comparison of H3K4me3 and H3K27me3 marks on dormancy regulators in WT seedlings and** ***fie*** **-seedlings based on microarray data from Bouyer et al., 2011.** Supplementary results to support data of **Fig. 4** **.** Upon loss of PRC2 activity in *fie*-mutants, the H3K4me3 mark stays on dormancy regulators through to the seedling stage.
(JPG)

Figure S4 **Histone H3 methylation pattern changes of regulators and markers of seed maturation/dormancy in Arabidopsis Cvi embryos of non-dormant seeds.** Supplementary results to support data of **Fig. 4** **.** Embryos were cleanly excised from seeds that had been subjected to 14 d of moist chilling. Data are based on the average of two biological replicates +/− S.D.
(JPG)

Table S1 **Primers used in this study.**
(DOC)
