# Correction: Evolutionarily Conserved Histone Methylation Dynamics during Seed Life-Cycle Transitions

**DOI:** 10.1371/annotation/cc0b3f20-ad10-4f1d-b238-548db29f5668

**Published:** 2013-12-13

**Authors:** Kerstin Müller, Daniel Bouyer, Arp Schnittger, Allison R. Kermode

Table 1, "Target genes of this study and their features." does not appear. Please view Table 1 here: http://plosone.org/corrections/pone.0051532.t001.cn.tif

