# Supplementary figures and images for: Evolutionarily Conserved Histone Methylation Dynamics during Seed Life-Cycle Transitions
Source: PLoS One. 2012 Dec 11;7(12):e51532. doi: 10.1371/journal.pone.0051532 (PMC3519861; doi:10.1371/journal.pone.0051532)

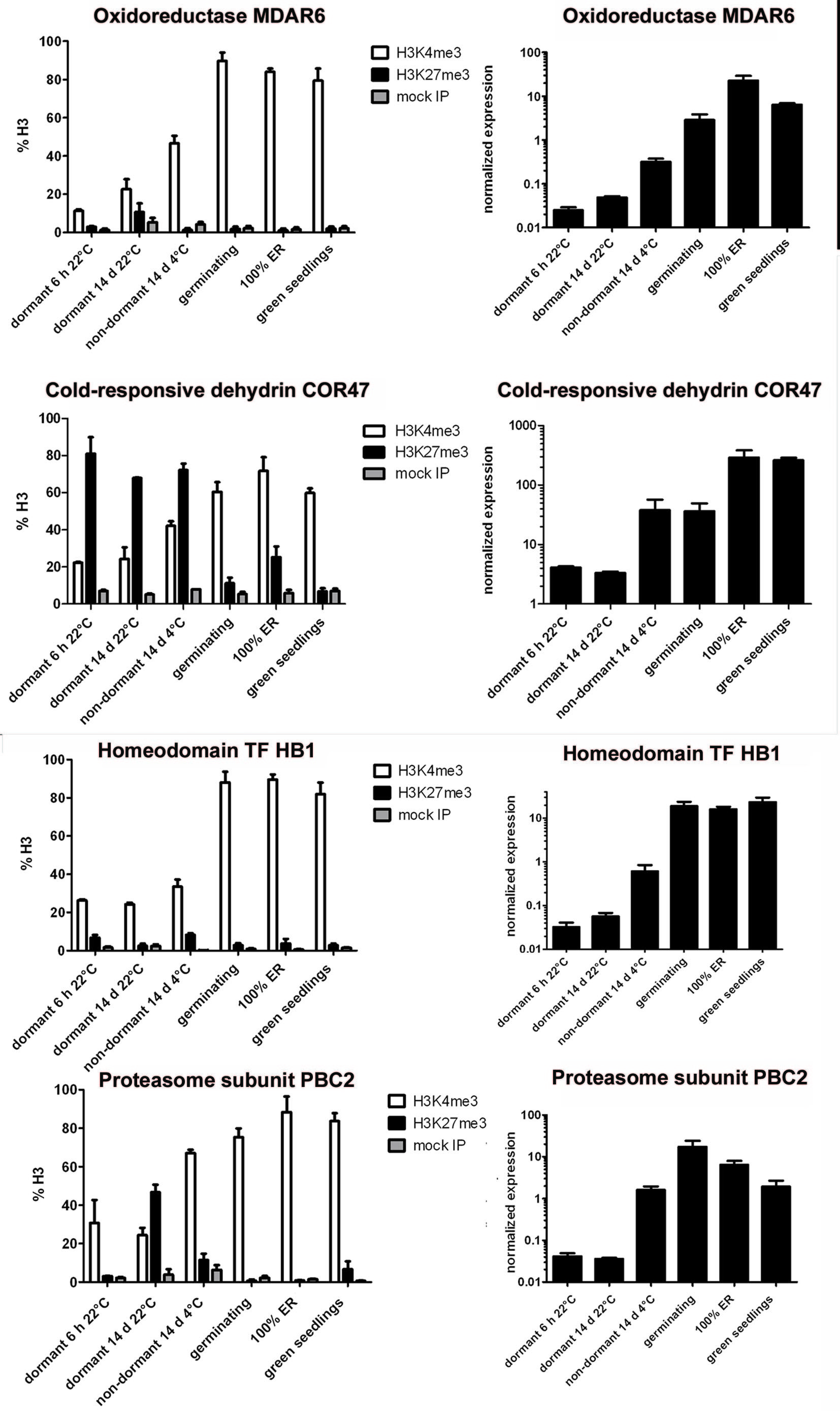

Supplement: Figure S1 — Expression analyses and histone H3 methylation pattern changes of regulators and markers of seed germination in Arabidopsis Cvi. Supplementary results to support data of Fig. 3 . nChIP/qPCR (left column) and expression analyses (right column); averages of three biological replicates are shown +/− SE. Refer to Table 1. ER = endosperm rupture and radicle emergence (completion of germination). Note that the Y-axis for the RNA data is in log-scale. (JPG) [file pone.0051532.s001.jpg]

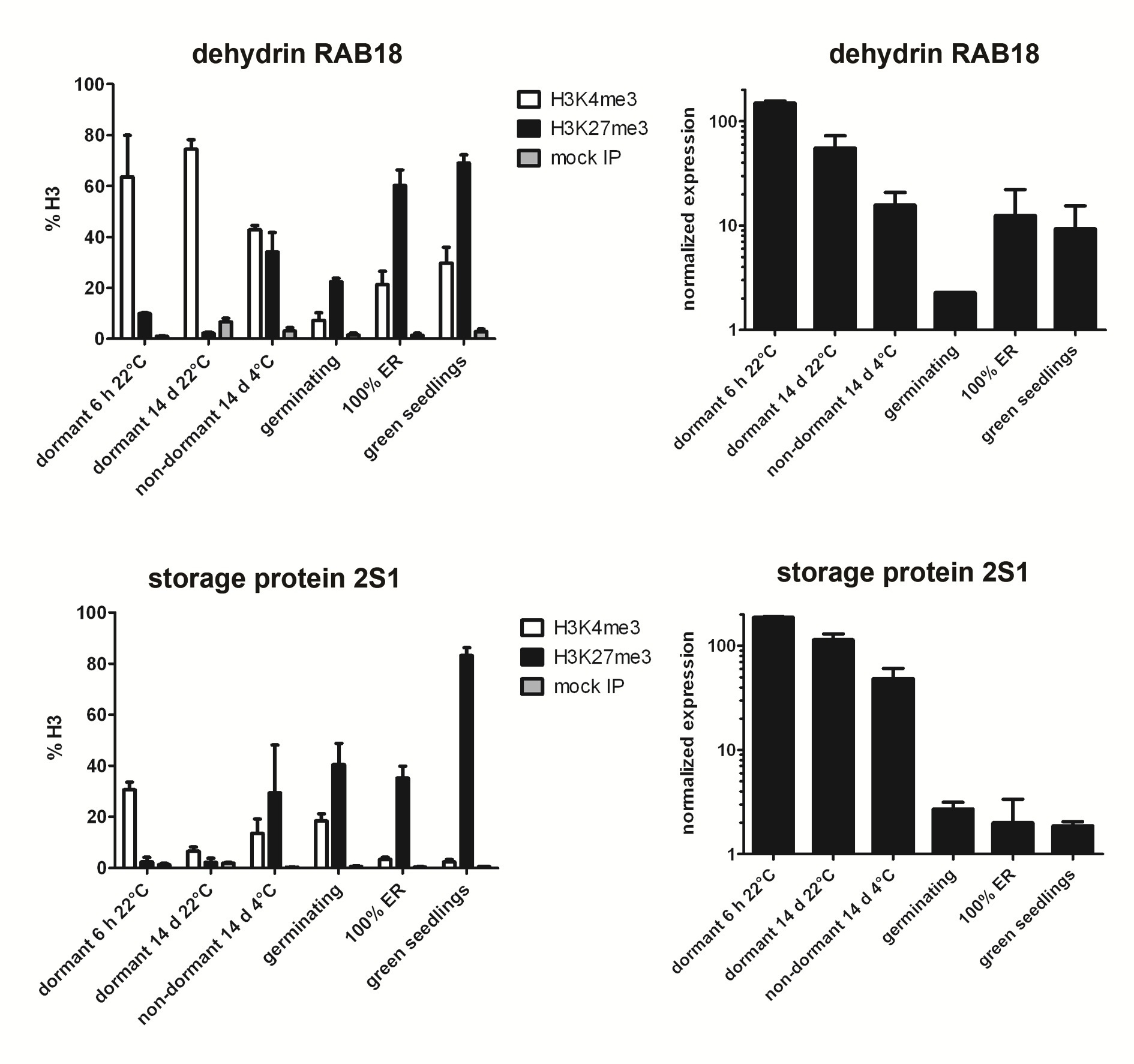

Supplement: Figure S2 — Expression analyses and histone H3 methylation pattern changes of markers of seed maturation/dormancy ( 2S1 and RAB18 ) in Arabidopsis Cvi. Supplementary results to support data of Fig. 4 . nChIP/qPCR (left column) and expression analyses (right column); averages of three biological replicates are shown +/− SE. Refer to Table 1. ER = endosperm rupture and radicle emergence (completion of germination). Note that the Y-axis for the RNA data is in log-scale. (JPG) [file pone.0051532.s002.jpg]

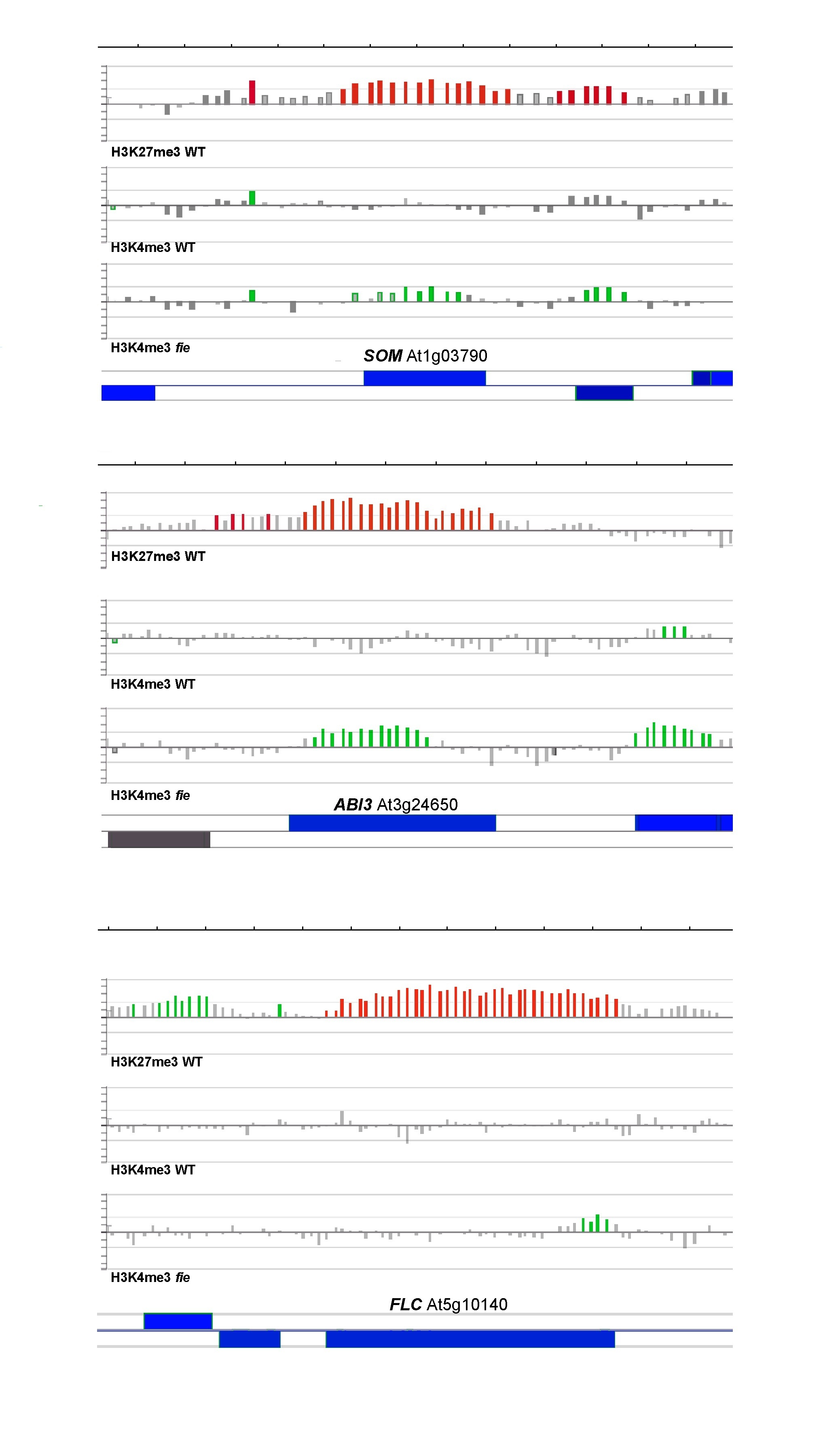

Supplement: Figure S3 — Comparison of H3K4me3 and H3K27me3 marks on dormancy regulators in WT seedlings and fie -seedlings based on microarray data from Bouyer et al., 2011. Supplementary results to support data of Fig. 4 . Upon loss of PRC2 activity in fie-mutants, the H3K4me3 mark stays on dormancy regulators through to the seedling stage. (JPG) [file pone.0051532.s003.jpg]

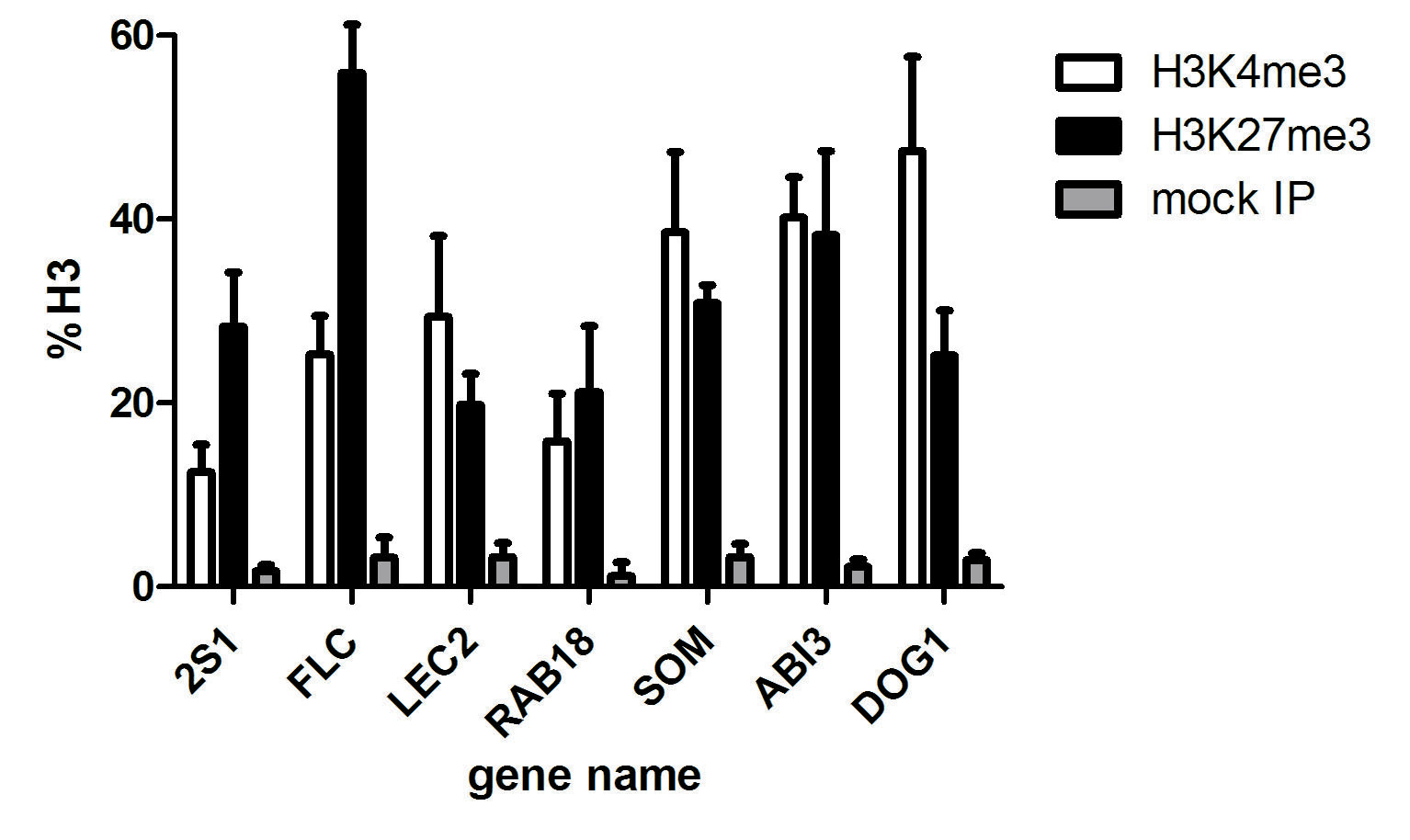

Supplement: Figure S4 — Histone H3 methylation pattern changes of regulators and markers of seed maturation/dormancy in Arabidopsis Cvi embryos of non-dormant seeds. Supplementary results to support data of Fig. 4 . Embryos were cleanly excised from seeds that had been subjected to 14 d of moist chilling. Data are based on the average of two biological replicates +/− S.D. (JPG) [file pone.0051532.s004.jpg]
