# Supplementary material for: Evolutionarily Conserved Histone Methylation Dynamics during Seed Life-Cycle Transitions
Source: PLoS One. 2012 Dec 11;7(12):e51532. doi: 10.1371/journal.pone.0051532 (PMC3519861; doi:10.1371/journal.pone.0051532)
Supplement: Table S1 — Primers used in this study. (DOC) [file pone.0051532.s005.doc]

**Supporting Information**

**Evolutionarily conserved histone methylation dynamics during the dormancy-to-germination life-cycle transition in plants**

Kerstin Müller^1^, Daniel Bouyer^2^, Arp Schnittger^2^, Allison R. Kermode^1*^

^1^Department of Biological Sciences, Simon Fraser University, 8888 University Drive, Burnaby BC V5A 1S6, Canada.

^2^Institut de Biologie Moléculaire des Plantes du CNRS - UPR2357, 12 rue du Général Zimmer, 67084 Strasbourg, France

*Author for correspondence: Department of Biological Sciences, Simon Fraser University, 8888 University Drive, Burnaby BC V5A 1S6, Canada. Email kermode@sfu.ca, phone +1-778-782-3982

| **Table S1. Primers used in this study.** | | |  |  |  |
| --- | --- | --- | --- | --- | --- |
|  |  |  |  |  |  |
| **Arabidopsis seed maturation/dormancy markers and regulators** | | | | |  |
| DOG1_for | GGCTCGTTTATTGGTCGAGGCCG | | | |  |
| DOG1_rev | TCCCAGCGAGCAAGAAATCCGC | | | |  |
| FLC_4for | TCCAAACGTCGCAACGGTCTCA | | |  |  |
| FLC_4rev | TAGAGCTTGCCGGAGGCGGA | | |  |  |
| FLC_27for | GCATGCTGATGATCTTAAAGCCTTGGT | | | |  |
| FLC_27rev | GCATGTGGAGCCACCACCTCATT | | | |  |
| LEC2_for | CGCTCGCACTTCACAACAGTCC | | |  |  |
| LEC2_rev | TCATCACCGCCGCCATCTGC | | |  |  |
| SOM_for | AAATGAGGAAGCCAACGGTGGAGA | | | |  |
| SOM_rev | TGTTTACGCGGCGAGCGAGG | | |  |  |
| RAB18_for | CCGTCCAGGAGGTCAGGCCA | | |  |  |
| RAB18_rev | AGCTCCTCCACCACCACCAGT | | |  |  |
| 2S1_for | CCTCGTCTGCGCAGCTCTCG | | |  |  |
| 2S1_rev | GGGCCTATGGGGTTAGTGGCG | | |  |  |
| AtCOR47_for | CCGAGCACGAGACACCAACGG | | |  |  |
| AtCOR47_rev | CGAACTCAGACTCGAGCGTCGT | | |  |  |
|  |  |  |  |  |  |
| **Arabidopsis seed germination and growth markers and regulators** | | | | |  |
| MBK20.1_for | GCGAAGGCCGTGGGGGAAAT | | |  |  |
| MBK20.1_rev | ACGGCTTTTCTTCCCCGGAGC | | |  |  |
| AtEXP10_for | GCACGGCGGCTCTAAGCACA | | |  |  |
| AtEXP10_rev | ACGCGTTATTTGGCGGGCAGA | | |  |  |
| MDAR6_for | GGTCGGCTATGCATTGTGACCAA | | | |  |
| MDAR6_rev | AGCCGGTCTCTCATAAGGTGCGT | | | |  |
| PBC2_for | AGTGATCGGAGGCTTGGTGTGC | | |  |  |
| PBC2_rev | CGGTGGCGAGACCGGAAAGT | | |  |  |
| AtHB1_for | AGGTGCATCTGCTGGAGAAAAGC | | | |  |
| AtHB1_rev | CAGCCACTTGCCTTGGCTGT | | |  |  |
|  |  |  |  |  |  |
| **Arabidopsis qRT-PCR reference genes** | | |  |  |  |
| At2g20000-qF1 | GTATAGCTCCACCACCACTT | | |  |  |
| At2g20000-qR1 | TCTTCTAGGTGCTTGAAGAGT | | |  |  |
| At1g17210-qF2 | CTGCTTCATATGAATCACGAG | | |  |  |
| At1g17210-qR2 | TCAACACTATCTGCACGTTGT | | |  |  |
|  |  |  |  |  |  |
| **Arabidopsis HMTs** | |  |  |  |  |
| ATX1_for | ATGTGCACCATCTGCGGGGT | | |  |  |
| ATX1_rev | TCGCGCGCAGAGTGGATGAT | | |  |  |
| ATX2_for | TGTGTTGCAAGACCGCCGCT | | |  |  |
| ATX2_rev | GGTCGCCGATGAGGCTGCAA | | |  |  |
| ATX4_for | TGTGTGCCTCCCGAGCTGGT | | |  |  |
| ATX4_rev | TCTGGGTTGGGAGCCCTGTGA | | |  |  |
| ATXR7_for | TGTCGTGCGGGTCAAGAGGC | | |  |  |
| ATXR7_rev | TGCTGTTGCGTGTAAGGGCCA | | |  |  |
| CLF_for | AGGCAAAGTGCGGCGCTTGA | | |  |  |
| CLF_rev | ACTGACGGCAGGGCTGGTCT | | |  |  |
| EZA1_for | TGTCCCTGCTTTGCTGCTGGC | | |  |  |
| EZA1_rev | TCCGCATTGCCCTTCTCCGC | | |  |  |
| MEA_for | AACATGCGGATTGGTCGCGTG | | |  |  |
| MEA_rev | ACGAGCTGGACGGGCTTCCT | | |  |  |
| ATX3_for | CGTGGCAGAGTTCGAGCGGT | | |  |  |
| ATX3_rev | CGACTCTCCCTTGCGACGGC | | |  |  |
| ATXR3_for | GCGGGTGACCAAATTGGCGG | | |  |  |
| ATXR3_rev | TGCATGCGACACCCCCATCG | | |  |  |
|  |  |  |  |  |  |
| **yellow-cedar CnABI3** | |  |  |  |  |
| CnABI3_for | CAAGCAGAGCGACGTGGGCA | | |  |  |
| CnABI3_rev | GCCTCCAGCTCAGGAAGGTGC | | |  |  |
|  |  |  |  |  |  |
| **yellow-cedar qPCR reference gene** | | |  |  |  |
| YC18S_for | GTCTGCTTGGGCGTCGCACT | | |  |  |
| YC18S_rev | TCGACGGACCCCGTGCTCAT | | |  |  |
